# Bechterew's Phenomenon in Bilateral Sequential Vestibular Neuritis: A Report of Two Cases

**DOI:** 10.3389/fneur.2022.844676

**Published:** 2022-03-28

**Authors:** Yehree Kim, Siyeon Jin, Ji-Soo Kim, Ja-Won Koo

**Affiliations:** ^1^Department of Otolaryngology Head and Neck Surgery, Seoul National University College of Medicine, Seoul National University Bundang Hospital, Seongnam, South Korea; ^2^Department of Neurology, Seoul National University College of Medicine, Seoul National University Bundang Hospital, Seongnam, South Korea; ^3^Sensory Organ Research Institute, Seoul National University Medical Research Center, Seongnam, South Korea

**Keywords:** bilateral sequential vestibular neuritis, Bechterew's phenomenon, vibration-induced nystagmus, headshaking nystagmus, ocular torsion

## Abstract

The brain can compensate for the vestibular imbalance. When the unilateral labyrinthine function is lost, the asymmetry between the peripheral vestibular inputs is compensated centrally by readjusting the signal difference from both ears and regaining vestibular balance. If the other healthy labyrinth is destroyed, the vestibular nuclei become imbalanced again, creating spontaneous nystagmus even though there is no input to the vestibular nuclei from either labyrinth. This is called Bechterew's phenomenon; a rare and not widely recognized phenomenon that occurs in cases of bilateral sequential vestibular neuritis. This is of clinical importance because spontaneous nystagmus with bilaterally absent or diminished caloric responses may give a misleading impression of a central lesion rather than a second peripheral lesion superimposed upon the effects of central compensation for the first. Although well-documented in experimental animals, this phenomenon rarely occurs in human beings. The objective of this study is to highlight the characteristics and the progression of test results from two patients from our own experience. Along with careful history taking and physical examination, a complex interpretation of various vestibular function tests, including induced nystagmus, head impulse test, caloric test, and fundus photography, is needed to make an accurate diagnosis of bilateral sequential vestibular neuritis (BSVN).

## Introduction

Vestibular neuritis (VN) is a common peripheral vestibular disease, accounting for 7% of patients in a vertigo clinic (1, 2). The VN presents with acute vertigo that lasts over 24 h, spontaneous nystagmus that beats toward the unaffected ear, and a positive head impulse test. Resolution of the acute and severe rotatory vertigo ensues after 2–3 days in 70% of the patients, but, in 4%, it can last longer than 2 weeks (3, 4). The VN was traditionally known as a non-recurrent disease (5); however, reports of recurrence have been made (6).

Recurrence of VN can either be ipsilateral (7) or contralateral (5). Contralateral involvement of VN after completion of central compensation from initial VN is referred to as bilateral sequential vestibular neuritis (BSVN). The BSVN is a rare condition that has only been described in a limited number of publications (5, 6, 8–13). If the vestibular function from the first episode of VN is not recovered, the patient will develop spontaneous horizontal nystagmus beating away from the side involved during contralateral VN, while vestibular test results will show findings of bilateral vestibular hypofunction. These findings may give a misleading impression of central vertigo if the clinician is not aware of the first episode of unilateral VN that the patient has already gone through.

Bechterew first described this phenomenon after a series of surgical labyrinthectomies performed on animals (14); a pioneering work that revealed central compensation mechanisms that follow asymmetry of bilateral vestibular tones. Vestibular compensation readjusts the signal difference from both ears to regain vestibular balance; however, this dynamic process occurs at different rates to a different extent for different vestibular responses (15). For example, spontaneous nystagmus (static imbalance) resolves within days, whereas in post-head shaking nystagmus, nystagmus induced by position change and vibration-induced nystagmus (dynamic imbalance) persist, and their resolutions are less complete.

Earlier reports of BSVN are somewhat anecdotal (9, 10, 12, 13), but, with advancements in vestibular function tests, recent reports have provided a more in-depth description of the status of the vestibular periphery and the compensatory stage of patients with BSVN. To highlight the clinical characteristics of BSVN, we report 2 cases from our own experience. Especially, we discuss the progression of objective findings after the second insult in the contralateral ear using vibration-induced and headshaking nystagmus (HSN), video head impulse test (vHIT), and ocular torsion (OT) as seen on fundus photography, which, to our knowledge, is the first report of its kind in BSVN.

## Case Description

### Case 1

**Figure 1:**
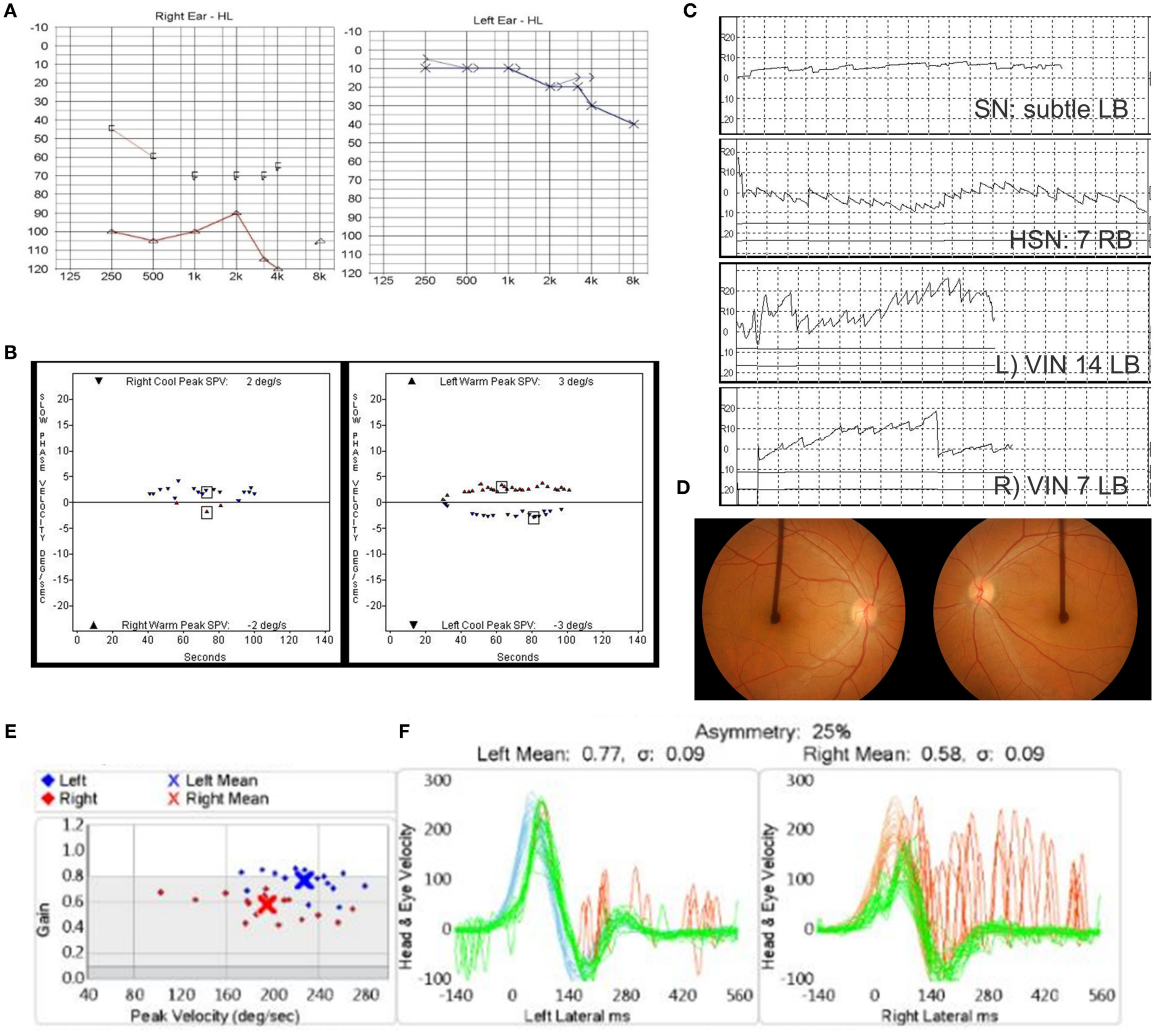
**(A–D)** Clinical assessment of Case 1 at 4 months after the onset of left vestibular neuritis. **(A)** Audiometric assessment shows right-side deafness. **(B)** The bithermal caloric test shows bilateral canal paresis. **(C)** Subtle left-beating spontaneous nystagmus, headshaking nystagmus (HSN) beating to the right, and vibration-induced nystagmus beating to the left. **(D)** Fundus photography revealed conjugate counterclockwise torsion from the viewpoint of the patient with extorsion of the right eye (4°) and increased extorsion of the left eye (17°). **(E)** The video head impulse test showed vestibulo-ocular reflex (VOR) gain of 0.58 on the right side and 0.77 on the left side 4 years later.

A 54-year-old female was presented to the clinic, complaining of oscillopsia and ataxia after sudden spontaneous vertigo accompanied by nausea and vomiting 4 months ago. Ten years previously, she reported another spell of spontaneous vertigo and hearing loss of the right ear. The vertigo spell resolved over time, but the hearing loss remained. Apart from the hearing loss, the patient did not recall other past or present medical conditions. Physical assessment revealed subtle right-beating spontaneous nystagmus under Frenzel glasses, a positive sign on impulse head rotation to both sides, no skew deviation, and no cerebellar signs. Audiometry showed right side deafness and age-appropriate hearing on the left with mild sensorineural hearing loss of the high frequencies (Figure 1A). The bithermal caloric test identified bilateral vestibular hypofunction. The sum of peak slow phase velocity (SPV) of warm and cold stimulation was 4°/s (right ear) and 6°/s (left ear) (Figure 1B). Cervical vestibular-evoked myogenic potentials (VEMPs) were bilaterally absent. Video nystagmography showed subtle left-beating spontaneous nystagmus (1°/s) at a sitting position. Headshaking evoked horizontal nystagmus beating to the right with a maximum SPV of 7°/s. A vibrator applied to the patient's left mastoid induced a horizontal nystagmus beating to the left with an SPV of 14°/s and left-beating nystagmus with an SPV of 7°/s when applied to the right mastoid (Figure 1C). The OT was assessed using fundus photography with a scanning laser ophthalmoscope (Fundus camera CF-60 UVI, Canon, Tokyo, Japan), with the patient's head upright. The OT was determined by measuring the angle between the horizontal line running through the center of the optic disc and a line connecting the center of the optic disc and fovea. A negative value of the angle indicates intorsion, and a positive value indicates extorsion. This patient revealed conjugate counterclockwise torsion from the viewpoint of the patient with extorsion of the right eye (4°) and increased extorsion of the left eye (17°) (Figure 1D). Brain MRI revealed a 7-mm lesion in the right frontal lobe, consistent with hemangioma, but no other noticeable findings in the posterior fossa or the temporal bone. A diagnosis of BSVN (the onset of the right side: 10 years ago, the onset of the left side: 4 months ago) was made, and she underwent vestibular rehabilitation. Four years later, the vHIT showed a VOR gain of 0.58 on the right side and 0.77 on the left side (Figure 1E). The sequential changes of the patient's vestibular function are summarized in Table 1.

**Table 1:** A summary of changes of vestibular function in case 1.

	**Right VN (10 years before left VN)**	**Left VN** **(4 months)**	**Left VN** **(1 year)**	**Left VN** **(4 years)**
SN (°/s)	NT	subtleLB	3 RB	0
HSN (°/s)	NT	7RB	9 RB	8 RB
VIN (°/s)	NT	R) 7 LB	R) 7LB	R) 18 LB
		L) 14 LB	L) 9 LB	L) 16 LB
Ocular	NT	R) 4°	R) 4°	NT
torsion (°)		L) 17°	L) 17°	
Caloric test	NT			
RW+RC (°/s)		4	5	0
LW+LC (°/s)		6	7	5
vHIT gain of	NT	NT	NT	R) 0.58
horizontal canal				L) 0.77
cVEMP	NT	R) NR	NT	NT
		L) NR		

*VN, vestibular neuritis; SN, spontaneous nystagmus; NT, not tested; RB, right beating; LB, left beating; HSN, headshaking nystagmus; VIN. Vibration-induced nystagmus; RW, right warm; RC, right cold; LW, left warm; LC, left cold; vHIT, video head impulse test; cVEMP, cervical vestibular evoked myogenic potentials; NR, no response*.

### Case 2

**Figure 2:**
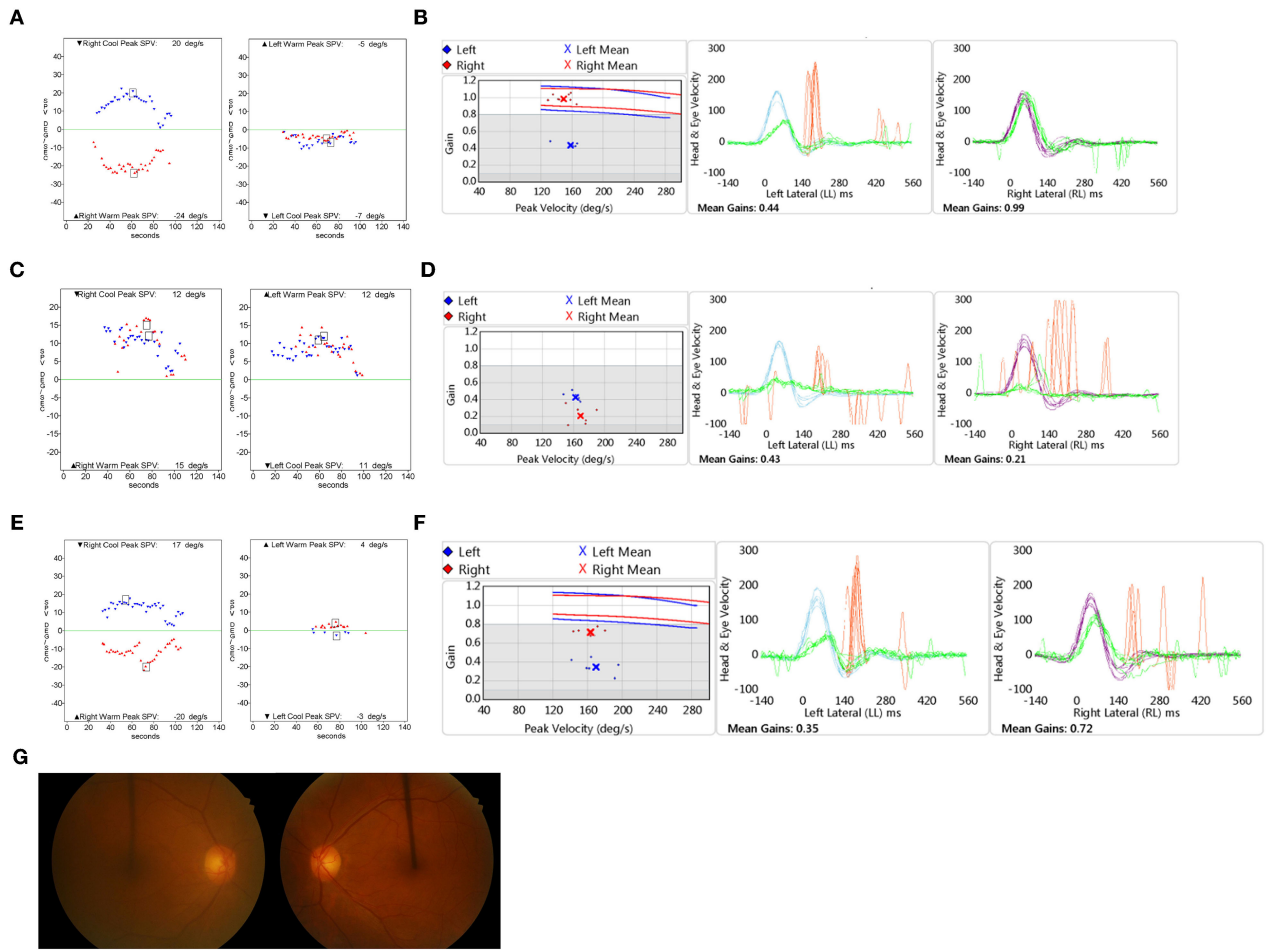
Clinical assessment of Case 2. The bithermal caloric test **(A)** and video head impulse test (vHIT) **(B)** results show unilateral vestibular hypofunction when the patient was diagnosed with left vestibular dysfunction initially. The bithermal caloric test **(C)** and vHIT **(D)** results show bilateral vestibular hypofunction when the patient was diagnosed with right vestibular dysfunction. The bithermal caloric test **(E)** and vHIT **(F)** performed 1 year later revealed partial recovery of the right side. **(G)** Fundus photography showed symmetric ocular torsion. vHIT: video head impulse test.

A 65-year-old male, with no underlying medical conditions, was presented to the emergency room with a 12-h history of severe acute rotatory vertigo (visual analog scale, VAS 10) without hearing impairment. Bed-side examination found right-beating spontaneous nystagmus under Frenzel glasses and showed a positive sign on the head impulse test to the left. Neurological evaluation revealed no skew deviation and no cerebellar signs. The bithermal caloric test revealed canal paresis of 91% of the left side (Figure 2A). vHIT showed decreased gain of the left lateral semicircular canal (Figure 2B). The patient was discharged with a diagnosis of left VN. Four years later, the patient returned to the emergency room complaining of another vertigo attack, which was milder in severity (VAS 8). Spontaneous nystagmus was beating to the left (12°/s), and the bedside head impulse test showed a positive sign in both directions. The bithermal caloric test revealed bilateral vestibular hypofunction. The sum of SPVs of warm and cold stimulation of right and left was −3 and 1°/s, respectively (Figure 2C). Substantial directional preponderance was shown due to strong spontaneous nystagmus. The vHIT showed markedly decreased gains of bilateral anterior and lateral semicircular canals (Figure 2D). A central cause was excluded by neurological examination, including the abovementioned head impulse, nystagmus, test of skew (HINTS), cerebellar function test, and a gadolinium-enhanced brain MRI. After corticosteroid treatment and vestibular rehabilitation, subjective discomforts of dizziness, imbalance, and oscillopsia were resolved over time. Four months later, headshaking evoked subtle horizontal nystagmus beating to the left with a maximum SPV of 1°/s. A vibrator was applied to the patient's left mastoid-induced horizontal nystagmus beating to the right with an SPV of 11°/s and right-beating nystagmus with an SPV of 8°/s when applied to the right. The bithermal caloric test showed partial restoration of canal response of the right side. The sum of SPVs of right warm and cold stimulation was 18°/s, while VOR gain of the right horizontal canal on vHIT was still 0.53. The sum of SPVs of left stimulation was 0. Fundus photography showed symmetric revealing extorsion of the right eye (4°) and extorsion of the left eye (5°). Cervical VEMPs were normal on both sides, whereas ocular VEMPs were bilaterally absent. Caloric response (Figure 2E) and VOR gain on vHIT of the right side after 1 year were further improved, while those of the left side was not much improved (Figure 2F). Fundus photography showed symmetric ocular torsion (Figure 2G). The sequential changes of the patient's vestibular function are summarized in Table 2. Both participants voluntarily participated in the study and provided written informed consent. This study was reviewed and approved by the Institutional Review Board.

**Table 2:** A summary of changes of vestibular function in Case 2.

	**Left VN (4 years ago)**	**Right VN** **(1 day)**	**Right VN** **(4 months)**	**Right VN** **(1 year)**
SN	8 RB	12 LB	0	0
HSN	20 RB	Not augmented	1 LB	0
VIN	R) 30 RB	NT	R) 8 RB	R) 19 RB
	L) 65 RB		L) 11 RB	L) 5 RB
Ocular	NT	NT	R) 4 ex	R) 5 ex
torsion			L) 5 ex	L) 6 ex
Caloric test				
RW+RC (°/s)	44	−3	18	37
LW+LC (°/s)	2	1	0	7
vHIT gain of	R) 0.99	R) 0.21	R) 0.53	R) 0.72
horizontal canal	L) 0.44	L) 0.43	L) 0.32	L) 0.35
cVEMP	NT	NT	Symmetric response	NT
oVEMP	NT	NT	Both no response	NT

*VN, vestibular neuritis; SN, spontaneous nystagmus; NT, not tested; RB, right beating; LB, left beating; HSN, headshaking nystagmus; VIN: vibration-induced nystagmus; RW, right warm; RC, right cold; LW, left warm; LC, left cold; vHIT, video head impulse test; cVEMP, cervical vestibular-evoked myogenic potentials; oVEMP, ocular vestibular-evoked myogenic potentials; NR, no response*.

## Discussion

The BSVN is a rare condition in which 1.9–5.3% of patients with unilateral VN develop later (1, 5, 8). During the first episode, the patients complained of severe whirling-type vertigo, whereas the main symptoms of the second episode were imbalance with slight vertigo. The direction of the spontaneous nystagmus of the first and second episodes changed. Although results of the vestibular function test showed bilateral hypofunction, spontaneous nystagmus was formed toward the side originally (the first episode) involved. This can be a rather strange and misleading clinical finding, raising the false suspicion of central nystagmus or cerebellar clamp, in which cerebellar inhibition suppresses the vestibular signal of the intact side to rebalance the asymmetry (16). However, the cerebellar clamp resolves within days after unilateral insult, a feature that is distinguishable from BSVN (17).

A phenomenon associated with Bechterew's nystagmus includes recovery nystagmus, which is spontaneous nystagmus beating toward the affected side observed temporarily for a few months after the initial insult (18). It results from persistent central compensation for an imbalance in vestibular tone after the need for this amount of compensation has diminished (19), and, possibly, restoration of end-organ function. Bechterew's phenomenon occurs because, after the central vestibular tone has been rebalanced following the first lesion, the second lesion creates a new imbalance (12).

In the 2 cases presented as our own experience, the ocular torsion on the fundus photography, HSN, and vibration-induced nystagmus were applied. In Case 1, the extorsion of the left eye (the side recently affected) was increased, and it was maintained over years, whereas the ocular torsion of the right eye was within a normal range for the patient's age (20). In Case 2, a fundus photograph was taken 4 months after the second ear attack. Unfortunately, the fundus photography was not taken immediately after the contralateral insult. If it was tested, increased extorsion to the newly affected side would be documented. The fundus photograph at 4 months after the second attack did not show asymmetric ocular torsion, which would be normalized with the gradual recovery of the end-organ function.

The HSN is generated by repetitive headshaking and usually beats toward the better ear in unilateral inhibitory vestibular lesions (19). The mechanism behind HSN is explained by Ewald's second law and the central velocity-storage system. During each head movement, the directionally asymmetric responses accumulate in the velocity-storage mechanism to be discharged after the head stops shaking (21). To interpret HSN, the condition of the velocity-storage mechanism must be taken into account (22). In BSVN, at the time of the second insult, the velocity-storage mechanism from the first insult would have recovered, thereby eliciting dynamic asymmetry of the recently affected side.

Vibration-induced nystagmus is not modified by vestibular compensation, so it is useful for differentiating bilateral areflexia. Patients with no caloric responses (a test for low frequencies) and decreased VOR gain for all 6 canals in vHIT (middle-range frequencies) show vibration-induced nystagmus due to residual hair cells still responding at high frequencies (100 Hz) (23).

## Conclusion

The BSVN is a rare condition but an interesting human model to understand the change of central compensation after deafferentation of peripheral vestibular input. To accurately diagnose BSVN and recognize current vestibular status, it is necessary to understand the pattern of induced nystagmus resulting from the central compensation mechanism and change of a peripheral vestibular function. A collective interpretation of the patient's history, as well as physical examination and comprehensive vestibular function tests, including headshaking and vibration-induced nystagmus, head impulse test, caloric test, fundus photography, is needed to understand and diagnose BSVN.

## Data Availability Statement

The original contributions presented in the study are included in the article/supplementary material, further inquiries can be directed to the corresponding author.

## Ethics Statement

The studies involving human participants were reviewed and approved by Seoul National University Bundang Hospital Institutional Review Board. The patients/participants provided their written informed consent to participate in this study. Written informed consent was obtained from the individual(s) for the publication of any potentially identifiable images or data included in this article. Written informed consent was obtained from the participants for the publication of this case report.

## Author Contributions

YK and J-WK designed the study and wrote the article. YK, SJ, J-SK, and J-WK collected and analyzed data. All authors read and approved the final manuscript.

## Conflict of Interest

The authors declare that the research was conducted in the absence of any commercial or financial relationships that could be construed as a potential conflict of interest.

## Publisher's Note

All claims expressed in this article are solely those of the authors and do not necessarily represent those of their affiliated organizations, or those of the publisher, the editors and the reviewers. Any product that may be evaluated in this article, or claim that may be made by its manufacturer, is not guaranteed or endorsed by the publisher.
